# Diagnosis of small bowel inflammation using small bowel capsule endoscopy combined with abdominal CT scan.

**DOI:** 10.1055/a-2560-4839

**Published:** 2025-04-04

**Authors:** Kentaro Ito, Tomoyoshi Shibuya, Hirotaka Ishino, Masashi Omori, Rina Odakura, Masao Kouma, Takafumi Maruyama, Kei Nomura, Osamu Nomura, Dai ishikawa, Akihito Nagahara

**Affiliations:** 1 12847 Department of Gastroenterology, Juntendo University, Bunkyo-ku, Japan

**Keywords:** Endoscopy Small Bowel, Capsule endoscopy, Small bowel endoscopy, Inflammatory bowel disease

## Abstract

**Background and study aims:**

Abdominal computed tomography (CT) scans are simple to perform and widely used in evaluating small bowel inflammation. However, detailed evaluation of small intestinal mucosa is difficult with CT. Conversely, small bowel capsule endoscopy (SBCE) is noninvasive and useful for evaluation of mucosal inflammation. We evaluated presence or absence of mucosal inflammation by SBCE in patients with CT findings of suspected small bowel inflammation and analyzed their backgrounds.

**Patients and methods:**

The Lewis score was determined by SBCE, and scores ≥ 135 placed 65 patients in the enteritis group and scores of < 135 placed 87 patients in the pseudoenteritis group.

**Results:**

Blood tests revealed higher C-reactive protein (CRP) levels in the enteritis group ( *P* < 0.01). Regarding comorbidities, chronic renal failure ( *P* < 0.01) and carcinoma ( *P* = 0.05) were more common in the enteritis group, as was use of proton pump inhibitors ( *P* = 0.02). Target sign, accordion sign, and fat stranding/centipede sign, which are known findings on CT of small intestinal inflammation, were more frequently observed in the enteritis group ( *P* < 0.01). Small intestinal wall thickness was greater in the enteritis group (5.3 mm vs 3.4 mm, *P* < 0.01) and the cut-off value was 4.15 mm

**Conclusions:**

Backgrounds of patients with inflammatory mucosa included high CRP, use of nonsteroidal anti-inflammatory medications, chronic renal failure, and cancer. If a patient with a thickened small intestinal wall (> 4.15 mm) on CT has these characteristics, it may be worth considering performing SBCE.

## Introduction

Small bowel inflammation comprises a group of diseases commonly encountered in routine gastroenterology practice. Prognosis depends on initiating specific treatment tailored to the etiology, but small bowel inflammation frequently presents with nonspecific clinical manifestations, making its cause difficult to identify. The most common causes are infectious diseases such as bacterial and viral infections. However, the differential diagnosis also includes Crohn's disease, drug-induced conditions such as those caused by nonsteroidal anti-inflammatory drugs (NSAIDs), inflammation associated with collagen diseases and vasculitis, allergic gastroenteropathy, and parasitic infections 1 .

**Fig. 1:**
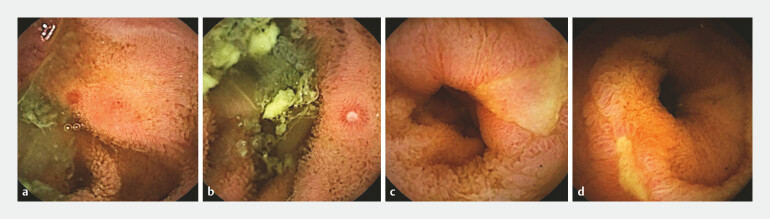
Findings of small bowel inflammation in SBCE. **a** Redness. **b** Erosion. **c,d** Ulcer.

Computed tomography (CT) is often the first imaging test performed in this context. However, many imaging findings are nonspecific, making evaluation of mucosal surfaces important for definitive diagnosis. Anatomical characteristics of the small bowel make the examination approach difficult, and for a long time, small bowel X-ray duplex contrast was the only means by which the entire small bowel could be viewed in relative detail. However, since 2000, capsule endoscopes and balloon-assisted endoscopes have become available, allowing for dramatic improvements in diagnosis of small bowel lesions. Small bowel capsule endoscopy (SBCE) offers significant advantages over previous techniques because it can noninvasively evaluate the entire small intestine. Because mucosal images are recorded, the small intestinal mucosa can be examined in detail ( Fig. 1 ). SBCE has been used to assess Crohn's disease and drug-induced enteritis, but there have been no standardized descriptions of the extent and severity of these lesions. In this context, in 2007, Gralnek et al. proposed the Lewis score to assess inflammation in the small intestine 2 . It provides an objective measure for monitoring. Scores < 135 are considered normal or indicative of clinically insignificant mucosal inflammatory changes, scores between 135 and 790 indicate mild inflammation, and scores ≥ 790 indicate moderate to severe inflammation.

Currently, there are no criteria for determining which cases require evaluation of the mucosal surface of the small intestine. This study evaluated the mucosal surface of the small intestine using SBCE in patients with suspected inflammatory changes in the small intestine determined by CT. We analyzed characteristics of cases in which inflammation of the mucosa was observed.

## Patients and methods

### Study design

This single-center retrospective study included 152 patients who underwent SBCE at our hospital between January 2014 and March 2024 and whose prior CT scans indicated inflammatory findings in the small intestine (small bowel edema, wall thickening, intestinal fluid retention, mesenteric lymphadenopathy, and ascites effusion). Medical records revealed data on patient background, comorbidities, medications, and CT findings. Simple and contrast CT scans were performed with patients in the supine position, and the imaging range was from the diaphragm to the pubis. Of the 152 patients, 48 underwent simple CT and 104 underwent contrast CT. SBCE was performed using a PillCam SB2/3, and RAPID for PillCam software was used to read the images (Covidien Japan Inc). This information was entered into a de-identified electronic database. The Juntendo University Ethics Committee (E24–0182) reviewed and approved this study, which was to be conducted in accordance with standards of good clinical practice and the principles of the Declaration of Helsinki.

### Patients

Images obtained by SBCE were analyzed and evaluated using the Lewis score. Patients with scores ≥ 135 were assigned to the enteritis group (N = 65) and those with scores < 135 were assigned to the pseudoenteritis group (N = 87).

### Statistical analysis

All data were analyzed with IBM SPSS Statistics, IBM Corp, Version 29.0. Differences between groups were determined by the Mann-Whitney’s U test and Fisher’s exact test. A significant difference was shown by *P* < 0.05.

## Results

### Patient characteristics

Table 1 shows characteristics of the 152 study patients (76 males and 76 females). Median age at examination was 54.4 years, median height was 162.7 cm, and median weight was 57.7 kg.

**Table:** **Table 1** Patient characteristics.

Sex, male/female		76/76		
Age (yr)*		54.4	(12–87)	
Height (cm)*		162.7	(134–188)	
BW (kg)*		57.7	(29–95)	
GTT (min)*		50.9	(1–508)	
SBTT (min)*		327.1	(55–749)	
Medicine	NSAIDs	20 (13.2%)	PPI	51 (33.6%)
	Antithrombotic	22 (14.5%)	MMP	23 (15.1%)
Co-morbid disease	Cardiovascular	18 (11.8%)	Collagen	18 (11.8%)
	Diabetes	15 (9.9%)	Thyroid	4 (2.6%)
	Hypertension	24 (15.8%)	Mental	2 (1.3%)
	CKD	16 (10.5%)	Carcinoma	27 (17.8%)
	CLD	22 (14.5%)	Abd surgery	16 (10.5%)
Abd, abdominal; BW, body weight; CKD, chronic kidney disease; CLD, chronic liver disease; GTT, gastric transit time; MMP, mucous membrane protectant; NSAID, nonsteroidal anti-inflammatory drug; PPI, proton pump inhibitor; SBTT, small bowel transit time. *Median.				

Median gastric transit time (GTT) was 50.9 minutes and median small bowel transit time (SBTT) was 327.1 minutes. Comorbidities included cardiovascular disease (18 patients), diabetes (15 patients), hypertension (24 patients), chronic kidney disease (CKD) (16 patients), chronic liver disease (22 patients), collagen disease (18 patients), thyroid disease (4 patients), psychiatric disease (2 patients), cancer (27 patients), and previous abdominal surgery (16 patients). No complications such as retention were observed during SBCE.

**Fig. 2:**
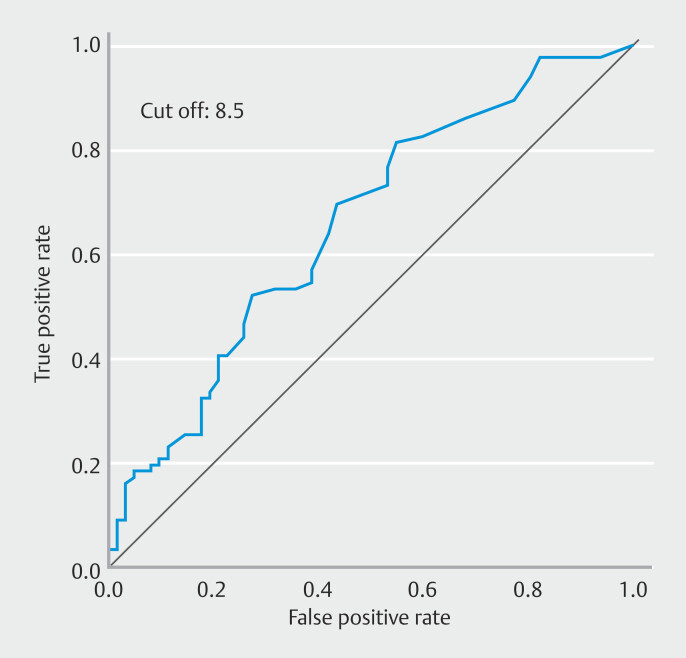
Analysis of average time from date of CT to date of SBCE. Logistic regression analysis determined the cutoff value to be 8.5 days (AUC 0.657).

Average time from date of CT to date of SBCE was 26.4 days (range 1–128). Average time in the enteritis group was 19.0 days and average time in the pseudo-enteritis group was 31.7 days. The enteritis group had a significantly shorter time ( *P* < 0.01). Logistic regression analysis was used to draw a receiver operating characteristic curve (ROC). The area under the ROC curve (AUC) was 0.657 and the cutoff value was 8.5 days when the Youden index was used to calculate it ( Fig. 2 ).

Medications taken were NSAIDs (20 patients), antithrombotic drugs (22 patients), proton pump inhibitors (PPIs) (51 patients), and mucous membrane protectant drugs (23 patients). There were no significant differences between the enteritis and pseudoenteritis groups in sex ratio, age, height, or weight ( Table 2 ). M\median Lewis score for the enteritis group was 451 (range 135–2140).

**Table:** **Table 2** Analysis of patient characteristics, GTT, SBTT, and blood findings.

	Enteritis:pseudoenteritis	*P* value
Sex (male/female)	30/35:46/41	0.52
Age	55.4:53.8	0.62
Height (cm)	163.1:162.4	0.65
BW (kg)	55.6:59.2	0.16
GTT (min)	40.9:58.2	0.37
SBTT (min)	147.7:129.5	0.47
WBC (/µL)	5985:6067	0.86
CRP (mg/dL)	1.57:0.69	0.01
BW, body weight; CRP, C-reactive protein; GTT, gastric transit time; SBTT, small bowel transit time; WBC, white blood cell.		

### Examination results

There were no significant differences in white blood cell (WBC) counts between the two groups nor were there significant differences in GTT or SBTT. C-reactive protein (CRP) level (mg/dL) was significantly higher in the enteritis group (1.57 vs. 0.69, *P* = 0.01).

### Medications

As shown in Table 3 , there were no significant differences in use of NSAIDs, antithrombotic drugs, or mucous membrane protectants between the two groups. PPIs were used significantly more frequently in the enteritis group (28 vs. 23 patients, *P* = 0.02).

**Table:** **Table 3** Analysis of oral medications.

Medication	Enteritis:pseudoenteritis	*P* value
NSAIDs	12:8	0.08
Antithrombotic drug	10:12	0.48
PPI	28:23	0.02
MMP	10:13	0.11
MMP, mucous membrane protectant; NSAID, nonsteroidal anti-inflammatory drug; PPI, proton pump inhibitor.		

### Comorbidities

Analysis of comorbidities ( Table 4 ) among patients showed no significant differences between groups in cardiovascular disease, diabetes, hypertension, chronic liver disease, collagen disease, thyroid disease, psychiatric diseases, or history of abdominal surgery. However, CKD and carcinomas were significantly more common in the enteritis group (15 vs. 1, 16 vs. 11; *P* = 0.01 and *P* = 0.05, respectively). Among patients in the enteritis group, there were two gastric cancers, two colorectal cancers, two pancreatic cancers, three liver cancers, two lung cancers, one prostate cancer, and four hematologic cancers. Among patients in the pseudoenteritis group, there was one gastric cancer, three pancreatic cancers, two liver cancers, three lung cancers, one ovarian cancer, and one hematologic cancer. There was no significant difference in cancer types between the two groups ( Table 5 ). Twelve patients (18.5%) in the enteritis group and four (4.6%) in the pseudoenteritis group received antitumor drugs, a finding that was significantly higher in the enteritis group ( *P* = 0.01). All patients in the enteritis group were suspected of having small intestinal symptoms and underwent CT scans, with three patients presenting with anemia, four with diarrhea and abdominal pain, and five with gastrointestinal hemorrhage. Regimens administered in the enteritis group included gemcitabine and nab-paclitaxel in two patients, aclarubicin and cytarabine (CA) in two patients, modified LSG15 in one patient, docetaxel in one patient, azacitidine in one patient, hepatic infusion therapy (fluorouracil and cisplatin) in one patient, durvalumab in two patients, and pembrolizumab in two patients.

**Table:** **Table 4** Analysis of co-morbid disease.

Co-morbid disease	Enteritis:pseudoenteritis	*P* value
Cardiovascular	11:7	0.08
Diabetes	6:9	0.52
Hypertension	14:10	0.07
CKD	15:1	< 0.01
CLD	13:9	0.75
Collagen	10:8	0.18
Thyroid	3:1	0.21
Mental	1:1	0.67
Carcinoma	16:11	0.05
Abdominal surgery	9:7	0.19
CKD, chronic kidney disease; CLD, chronic liver disease.		

**Table:** **Table 5** Analysis by cancer type.

Cancer	Enteritis	Pseudoenteritis	*P* value
Gastric	2	1	0.58
Colorectal	2	0	0.18
Pancreatic	2	3	1.0
Liver	3	2	0.65
Lung	2	3	1.0
Prostate	1	0	0.43
Ovarian	0	1	1.0
Blood	4	1	0.17

### CT findings

**Fig. 3:**
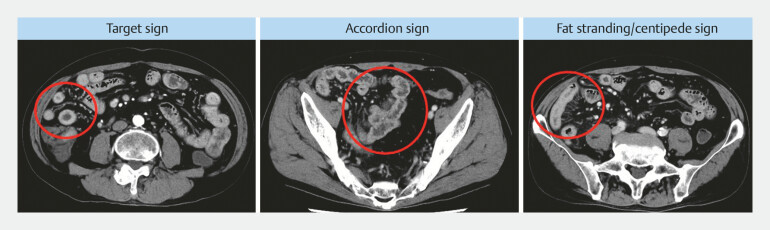
Findings of small bowel inflammation on CT. **a** Target sign. **b** Accordion sign. **c** Fat stranding/centipede sign.

**Fig. 4:**
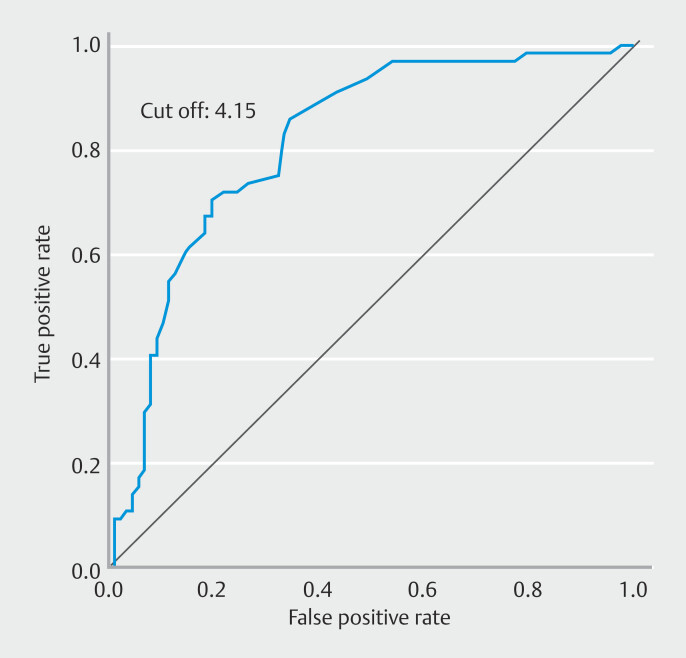
Analysis of small bowel wall thickness to predict the presence of small bowel inflammation. Logistic regression analysis determined the cutoff value to be 4.15 mm (AUC 0.814).

Reasons for CT examinations were abdominal pain/diarrhea in 49 patients (32.2%), anemia in 50 patients (32.9%), inflammatory bowel disease in 13 patients (8.6%), search for malignancy in 39 patients (25.7%), and post-ileus evaluation in one patient (0.7%). Thickness of the small intestinal wall (mm) was greater in the enteritis group than in the pseudoenteritis group (5.3 vs. 3.4, *P* < 0.01). Also, the target, accordion, and fat stranding/centipede signs ( Fig. 3 ) were significantly more common in the enteritis group (43 vs. 20, 29 vs. 17, 44 vs. 33, *P* < 0.01, *P* < 0.01) ( Table 6 ). Using logistic regression analysis to examine the relationship between small bowel wall thickness in the enteritis and pseudoenteritis groups, drawing an ROC curve and determining the optimal small bowel wall thickness using the Youden index yielded an AUC of 0.814 with a cutoff value of 4.51 mm ( Fig. 4 ).

**Table:** **Table 6** Analysis of CT findings.

CT findings	Enteritis:pseudoenteritis	*P* value
Wall thickness (mm)	5.3:3.4	< 0.01
Target sign	43:20	< 0.01
Accordion sign	29:17	< 0.01
Fat stranding/centipede sign	44:33	< 0.01
CT, computed tomography.		

## Discussion

We showed that SBCE in patients with suspected small bowel inflammation on CT differentiated true cases of enteritis by assessing the mucosal surface. Sixty-five patients (42.8%) were in the enteritis group.

No significant between-group differences were noted in age, gender, height, or weight. Comparisons of WBC values and CRP, which indicate inflammation, showed significantly higher CRP values in the enteritis group ( *P* = 0.01). Specific laboratory findings by SBCE included GTT and SBTT, with GTT usually ranging from a few minutes to 12 minutes. Although prolonged GTT has not been clearly defined, Freitas et al. defined prolonged GTT according to requirements for interventions such as placement of a capsule. They noted older age, being female, and having diabetes mellitus and/or psychiatric disorders (indicated by use of psychotropic drugs) as risk factors for prolonged GTT 3 . In the present study, median GTT was 14.5 minutes, with no significant difference between the two groups; 18 patients (11.9%) had prolonged GTT. There were no significant differences in age, gender, or presence of diabetes or psychiatric disorders. Recent studies have suggested that longer SBTT is associated with higher diagnostic yield from SBCE, with Arieira et al. reporting prolonged SBTT with findings such as ulcers, vasodilation, and tumors (interpreted as indicating that longer transit time through the small bowel increases likelihood of detecting significant findings) 4 . Mohan et al. reported that 220 minutes is sufficient transit time to accurately locate hemorrhage, if present 5 . In the present study, median SBTT tended to be longer in the enteritis group than in the pseudoenteritis group, but the difference was not statistically significant: 147.7 min vs. 129.5 min.

Previous reports have showed that small intestinal mucosal lesions in CKD patients were generally found in 20% to 30% of cases, and vascular lesions such as angiodysplasia tended to be more common 6 . CKD has also long been known as a risk for obscure gastrointestinal bleeding and SBCE is often used to assess for bleeding, because the source of bleeding is seldom identified on upper or lower endoscopy. For example, Kawamura et al. performed SBCE in 42 CKD patients (19 maintenance dialysis and 23 non-dialysis) and reported that mucosal lesions such as ulcers and erosions were observed in 14 patients (33.3%) and vascular lesions in 20 patients (47.6%) 7 . In the present study, 16 patients had CKD as a comorbidity (grade 2: 2 patients, grade 3: 8 patients, grade 4: 3 patients, grade 5: 3 patients). Maintenance dialysis was performed in the three patients with grade 5. There were significantly more patients in the enteritis group that had CKD complications than in the pseudoenteritis group (15 vs. 1, *P* < 0.01). Although many previous reports have described mucosal disorders in patients with severe CKD and those on maintenance dialysis 6 7 , the current results were notable in that mucosal damage was also observed in patients with relatively mild CKD (grades 2 and 3). The purpose of the SBCE examination was to investigate gastrointestinal bleeding in nine cases (56.3%), which was the most common reason among the study participants. Noting that there were significantly more patients with carcinoma in the enteritis group than in the pseudoenteritis group, whether there were between-group differences according to carcinoma was examined but no significant differences were found. We reviewed previous reports about the association between cancer and small intestinal enteritis, but were unable to find any notable information. Consequently, we conducted an additional analysis regarding use of anticancer drugs, and the results indicated a significant increase in their use in the enteritis group. The potential role of anticancer drugs in onset of enteritis has been discussed in previous studies. Due to their cytotoxic properties, antitumor drugs are more likely to damage organs with rapid turnover, such as skin and mucous membranes. For taxanes, necrosis of the epithelium and ulcer formation have been observed, with reports of gastritis and duodenal necrosis in mice and colon necrosis in dogs 8 . Adverse events in humans include vomiting and diarrhea, with risk of intestinal perforation, gastrointestinal bleeding, and colitis. Similarly, reports of enteritis caused by 5-fluorouracil (5-FU) have been recognized 9 , and it is possible that carrimycin therapy, a cytotoxic anticancer treatment similar to 5-FU, also caused damage to intestinal mucosa. In addition, three of four patients who were evaluated with SBCE and who had received immune checkpoint inhibitors were diagnosed with immune-related adverse events (irAE). These results suggest that administration of antitumor drugs, rather than presence of carcinoma, may have contributed to development of enteritis.

Although NSAIDs are well-known causes of disorders of the gastric and duodenal mucosa, there have also been reports about development of small intestinal mucosal injuries using capsule endoscopy 10 11 . Although we found no significant differences between the two groups, there were 12 cases (18.5%) of mucosal injuries in the enteritis group and eight cases (9.2%) in the pseudoenteritis group, with more internal injuries in the enteritis group ( *P* = 0.08). PPIs are useful in preventing gastric and duodenal ulcers due to NSAIDs, but a very high incidence of small intestinal mucosal damage has been reported in patients who took both drugs orally for 2 weeks (55%-75% in the treated group vs. 7%-11% in the placebo group) 12 . Microscopic colitis (collagenous colitis) is a widespread side effect of PPIs 13 , but there have also been several reports of small bowel mucosal damage. John et al. reported a significant increase in small intestinal damage in PPI-treated mice and postulated that PPIs reduced bactericidal capacity of the stomach due to their potent acid-suppressive effect, which leads to changes in intestinal microbiota and contributes to mucosal damage 14 . Fujimori et al. reported development of small intestinal mucosal damage in healthy study participants treated with PPIs for 2 weeks 15 . In the present study, PPI oral administration was noted in 28 patients (43%) in the enteritis group and 23 (26.4%) in the pseudoenteritis group, suggesting that oral administration of PPIs was significantly more common in the enteritis group ( *P* = 0.02). It may be a contributing factor in development of small intestinal inflammation.

Various CT findings are suggestive of small bowel inflammation, including small bowel edema, bowel wall thickening, intestinal fluid accumulation, mesenteric lymphadenopathy, and ascites effusion. However, all are nonspecific, and currently, there are no clear diagnostic criteria for small intestinal inflammation. The normal intestinal wall thickness of the small intestine was reported to be 1 to 2 mm and ≤ 3 mm or less in the colon when the lumen is fully dilated, with variations depending on degree of distension and presence of contents 16 . Wall thicknesses of 2 to 3 mm in the small intestine and around 4 mm in the colon are often interpreted as the upper limit of normal 17 18 . In this study, median thickness of the small bowel wall was significantly thicker in the enteritis group compared with the pseudoenteritis group: 5.3 mm vs. 3.4 mm ( *P* < 0.01). The cutoff value was determined as 4.15 mm (AUC 0.814) using the Youden index ( Fig. 4 ). In addition to these findings, several CT findings suggestive of small intestinal inflammation have been reported. The target sign indicates edema in the submucosa due to increased blood flow and hyperemia caused by inflammation and generally suggests a benign inflammatory disease. The accordion sign indicates narrowing of the intestinal lumen due to swelling of the haustra caused by inflammation. Initially observed after administration of a positive contrast agent, it may also be recognized as a state of intestinal folding with contrast effects. This sign is particularly common in infections characterized by significant wall thickening, such as pseudomembranous enteritis and cytomegalovirus enteritis, in which mucosal injury is intense but nonspecific. The fat stranding/centipede sign indicates inflammatory changes in mesenteric adipose tissue and dilation of mesenteric vessels 18 19 . These signs were also significantly more common in the enteritis group ( *P* < 0.01) and may be useful in distinguishing true enterocolitis from false positives.

Presence or absence of double-balloon endoscopy (DBE) after SBCE was investigated in 16 participants in the enteritis group: three for nonspecific erosions/ulcers, five for Crohn's disease, two for Behçet's disease, one for eosinophilic enteritis, one for drug-induced enteritis, one for gastrointestinal stromal tumor (GIST), one for inflammatory polyps, and two for no particular abnormalities. Clinical diagnoses in the enteritis group participants who did not undergo DBE included 21 with small intestinal erosions/ulcers, seven with drug-induced enteritis, four with irAE, five with Crohn's disease, three with Behçet's disease, two with lupus enteritis, one with IgA vasculitis, one with Schoenlein-Henoch purpura, two with small bowel GIST, one with cytomegalovirus infection, one with anisakiasis infection, and one with extramural compression.

The number of patients who underwent DBE testing in the enteritis group was surprisingly low. The reason was that the majority of patients could be diagnosed and treated with SBCE alone. Of the 21 patients with erosion findings, six underwent DBE to investigate gastrointestinal bleeding. At the time of examination, there were no findings suggestive of active bleeding, and the patients were followed up. In the remaining 15 patients, changes were considered nonspecific and were followed up. For drug-induced enteritis and irAE, the causative agent was discontinued and treatment with steroids or molecularly targeted drugs was initiated. Disease-specific treatment was initiated for Crohn's disease, Behçet's disease, lupus enteritis, IgA vasculitis, and Schoenlein-Henoch purpura. Cytomegalovirus infection was treated with antiviral drugs and small bowel GISTs were treated with surgery. In one case of extramural compression, surgery was performed for suspicion of a mesenteric tumor in conjunction with CT results (pathology showed a lymph follicle, with no malignant findings). Thus, in 49 participants (75.4%) in the enteritis group, treatment decisions were made based on SBCE findings. Regarding the Lewis score in the pseudoenteritis group, 79 patients scored 0 points, five patients scored 112 points, and three patients scored eight points. With respect to SBCE findings, 52 of 87 cases were deemed normal. Among the abnormal findings, 10 cases were suggestive of submucosal tumors or polyps, and 10 cases exhibited lymphoid hyperplasia and white villi. In addition, capillary dilation and mild gastrointestinal bleeding were observed in eight patients, whereas findings indicative of mild small intestinal inflammation (Lewis score < 135) were present in seven patients. For cases suspected of submucosal tumors, further evaluation was performed using a small intestine endoscope, or surgical treatment was undertaken. Among cases suggestive of gastrointestinal bleeding, two showed active bleeding in the small intestine. However, due to advanced age and reduced ability to perform activities of daily living in these patients, they were managed with blood transfusions and supportive care. Most of the remaining patients with minor findings were closely monitored.

Our study has several limitations. It was single-center and retrospective. Small bowel wall thickness and target, accordion, and fatty chain crossing/centipede signs were assessed by gastroenterologists alone, and the CT scan protocol was left to the attending physician and the examining physician, and no specific protocol was set. In some cases, information about the characteristics of intestinal inflammation may have been absent because of the long interval between CT scan and SBCE. Multivariate analyses were conducted with factors that differed significantly in each analysis, but no factor showed a significant difference. In the future, it is hoped that further cases will be accumulated and that prospective studies will be conducted in collaboration with radiologists.

## Conclusions

Inflammatory mucosa was associated with high CRP levels, use of PPIs, coexisting chronic renal failure, use of antitumor drugs and small bowel wall thickening (> 4.15 mm) shown on CT. If a patient with suspected small intestinal inflammation on CT has these characteristics, it may be worth considering performing SBCE.
